# Supplementary material for: Symptom evolution following the emergence of maize streak virus
Source: eLife. 2020 Jan 15;9:e51984. doi: 10.7554/eLife.51984 (PMC7034976; doi:10.7554/eLife.51984)
Supplement: Supplementary file 1. [file elife-51984-supp1.docx]

**Supplementary File 1.** Table: Regression analysis of mean inferred vs observed symptom trait values.

| **Symptom** | **Pearson R^2^** | **Slope** | **Average Pagel’s λ** | **p-value** |
| --- | --- | --- | --- | --- |
| Chlorotic area | 0.58 | 0.85 | 0.68 | 6.1 x 10^-4^ |
| Intensity of chlorosis | 0.18 | 0.40 | 0.53 | 2.7 x 10^-2^ |
| Leaf deformation | 0.09 | 0.15 | 0.36 | 1.8 x 10^-1^ |
| Leaf stunting | 0.73 | 0.86 | 0.60 | < 1 x 10^-5^ |
