# Supplementary material for: Symptom evolution following the emergence of maize streak virus
Source: eLife. 2020 Jan 15;9:e51984. doi: 10.7554/eLife.51984 (PMC7034976; doi:10.7554/eLife.51984)
Supplement: Supplementary file 2. [file elife-51984-supp2.docx]

**Supplementary File 2.** Table: Sites within ancestral sequences that could not be determined with a high degree of confidence (sites with a posterior probability of <0.8 in all attempted ancestral sequence inferences).

| **Ancestor** | **Alignment File** | **Site number** | **Inferred state (p)** | **Best alternative (p)** |
| --- | --- | --- | --- | --- |
| A0 | SI File 1 | 60  1120  1463  1975  2313  2616  2721  2753 | T (0.79)  G (0.57)  T (0.69)  G (0.68)  T (0.66)  A (0.79)  C (0.79)  T (0.79) | C (0.19)  C (0.38)  C (0.31)  T (0.31)  A (0.33)  G (0.11)  G (0.18)  C (0.19) |
| A1 | SI File 1 | 107  184  1416  1421  1435  1439  1784  1826  2620  2706  2709 | C (0.64)  C (0.71)  A (0.60)  A (0.59)  G (0.62)  G (0.61)  G (0.46)  G (0.68)  C (0.58)  A (0.76)  T (0.52) | T (0.34)  T (0.29)  G (0.39)  G (0.41)  C (0.38)  T (0.38)  T (0.43)  T (0.32)  T (0.42)  T (0.24)  C (0.48) |
| A2 | SI File 2 | 1317  2589  2614  2617  2654  2661  2673  2685  2715  2733  2768  2772  2781  2784  2785 | A (0.65)  T (0.78)  C (0.77)  A (0.77)  G (0.79)  G (0.75)  G (0.77)  C (0.77)  T (0.76)  C (0.74)  A (0.73)  C (0.69)  T (0.69)  A (0.79)  G (0.79) | G (0.34)  G (0.09)  A (0.10)  G (0.10), T(0.10)  T (0.11)  T (0.14)  A (0.11), T (0.11)  A (0.11), T (0.11)  C (0.12), G (0.12)  G (0.13). T (0.13)  C (0.13), T (0.13)  A (0.14), T (0.14)  A (0.17)  C (0.17)  A (0.17) |
| A3 | SI File 2 | 246  1136  1944  2754  2771  2779 | A (0.80)  T (0.79)  T (0.80)  C (0.71)  C (0.79)  G (0.73) | G (0.14)  A (0.15)  G (0.14)  A (0.12). T (0.12)  A (0.14)  A (0.12), T (0.12) |
| A4 | SI File 3 | 58  1013  2447  2654  2781  2783  2784  2785 | A (0.63)  A (0.73)  A (0.58)  C (0.65)  T (0.79)  A (0.80)  T (0.79)  A (0.79) | C (0.37)  G (0.26)  C (0.33)  T (0.25)  G (0.21)  C (0.16)  C (0.17)  T (0.17) |
| A5 | SI File 2 | 2589  2614  2617  2621  2654  2661  2673  2685  2706  2715  2733  2768  2772  2781  2783  2784  2785 | C (0.78)  C (0.77)  C (0.77)  T (0.78)  A (0.75)  C (0.75)  T (0.77)  A (0.77)  T (0.62)  C (0.76)  A (0.74)  T (0.73)  T (0.69)  A (0.69)  C (0.80)  T (0.79)  T (0.79) | A (0.09), T (0,09)  A (0.10), T (0.10)  A (0.10), T (0.10)  G (0.17)  T (0.11)  T (0.14)  A (0.11), C (0.11)  C (0.11), T (0.11)  C (0.24)  G (0.12). T (0.12)  C (0.13), T (0.13)  G (0.14), T (0.14)  A (0.14). G (0.14)  T (0.17)  T (0.16)  C (0.16)  A (0.16) |
| A6 | SI File 2 | 2589  2597  2614  2617  2661  2733  2768  2772  2781 | A (0.80)  C (0.80)  T (0.78)  C (0.78)  T (0.77)  C (0.79)  A (0.77)  A (0.71)  C (0.71) | G (0.08), T (0.08)  T (0.11)  A (0.09), G (0.09)  A (0.09), T (0.09)  C (0.13)  G (0.11), T (0.11)  C (0.11), T (0.11)  G (0.11), T (0.11)  T (0.16) |
