# Supplementary material for: Symptom evolution following the emergence of maize streak virus
Source: eLife. 2020 Jan 15;9:e51984. doi: 10.7554/eLife.51984 (PMC7034976; doi:10.7554/eLife.51984)
Supplement: Supplementary file 3. [file elife-51984-supp3.docx]

**Supplementary File 3**. Table: MSV isolates for which symptoms were quantified.

| **Accession #** | **MSV isolate** | **Sampling year** |
| --- | --- | --- |
| FJ882115 | MSV-A [ZA_Hei_O9_1979] | 1979 |
| FJ882132 | MSV-A [ZA_Pot4_O28_1979] | 1979 |
| FJ882134 | MSV-A [ZA_Pot6_O29_1979] | 1979 |
| FJ882136 | MSV-A [ZA_Pot8_O33_1979] | 1979 |
| FJ882131 | MSV-A [ZA_Pot3_O27-1979] | 1979 |
| X01633 | MSV-A [NG_Ns_1980] | 1980 |
| FJ882140 | MSV-A [ZW_Har2_Mic22_1987] | 1987 |
| KY618107 | MSV-A [ZA_Gre_O59_1987] | 1987 |
| KY618115 | MSV-A [ZA_Bet_O60_1987] | 1987 |
| KY618117 | MSV-A [ZA_Bet_O65_1987] | 1987 |
| KY618100 | MSV-A [ZA_Let_O83_1987] | 1987 |
| KY618102 | MSV-A [ZA_Let_O87_1987] | 1987 |
| KY618105 | MSV-A [ZA_Let_O84_1987] | 1987 |
| HQ693421 | MSV-A [ZA_Mak2_M49_1988] | 1988 |
| KY618098 | MSV-A [ZA_Ila_O45_1988] | 1988 |
| AF003952 | MSV-A [ZA_Kom_1989] | 1989 |
| KY618086 | MSV-A [KE_Kim_O57_1990] | 1990 |
| KY618088 | MSV-A [KE_Ler_O54_1990] | 1990 |
| FJ882143 | MSV-A [ZW_Mas2_Mic4_1993] | 1993 |
| HQ693396 | MSV-A [RE_Pie4_Mic13_1994] | 1994 |
| AF329881 | MSV-A [ZW_MatA_1994] | 1994 |
| FJ882103 | MSV-A [RE_Jos1_Mic18_1995] | 1995 |
| AF329885 | MSV-A [KE_MtKA_1997] | 1997 |
| X94330 | MSV-A [RE_Reu2_1997] | 1997 |
| AF329878 | MSV-A [KE_Ama_1998] | 1998 |
| AF329884 | MSV-A [ZA_MakD_1998] | 1998 |
| AF329883 | MSV-A [ZW_MatC_1998] | 1998 |
| EF547117 | MSV-A [UG_Bug245_2005] | 2005 |
| EF547075 | MSV-A [UG_Bush53_2005] | 2005 |
| EF547099 | MSV-A [UG_Hoi154_2005] | 2005 |
| EF547081 | MSV-A [UG_Kab82_2005] | 2005 |
| EF547122 | MSV-A [UG_Kap292_2005] | 2005 |
| EF547096 | MSV-A [UG_Kib179_2005] | 2005 |
| EF547107 | MSV-A [UG_Kib188_2005] | 2005 |
| EF547087 | MSV-A [UG_Luw110_2005] | 2005 |
| EF547074 | MSV-A [UG_Mba41_2005] | 2005 |
| EF547083 | MSV-A [UG_Mpi_11_2005] | 2005 |
| EF547083 | MSV-A [UG_Mub94_2005] | 2005 |
| EF547079 | MSV-A [UG_Kas_75_2005] | 2005 |
| EF547085 | MSV-A [UG_Luw_107_2005] | 2005 |
| EF547064 | MSV-A [UG_Wak_4_2005] | 2005 |
| HQ693407 | MSV-A [ZA_Cat2_D3_2006] | 2006 |
| HQ693468 | MSV-A [ZW_Chi_Zim3_2006] | 2006 |
| FJ882141 | MSV-A [ZW_Hel2_Bet36_2006] | 2006 |
| FJ882142 | MSV-A [ZW_Mas1_Bet43_2006] | 2006 |
| EU628568 | MSV-A [ZA_ThoE_g132_2006] | 2006 |
| EU152254 | MSV-A [ZA_Nat_g195_2006] | 2006 |
| EU628575 | MSV-A [ZA_RosE_g131_2006] | 2006 |
| EU628576 | MSV-A [Zw_Nmg_g168_2006] | 2006 |
| HQ693340 | MSV-A [MZ_Bil6_Bet25_2007] | 2007 |
| HQ693342 | MSV-A [MZ_Chi1_chimoz_2007] | 2007 |
| HQ693346 | MSV-A [MZ_Inh2_Moz1_2007] | 2007 |
| HQ693358 | MSV-A [MZ_Map8_Moz3_2007] | 2007 |
| HQ693359 | MSV-A [MZ_Map9_Moz4_2007] | 2007 |
| HQ693363 | MSV-A [MZ_Pem2_Moz37_2007] | 2007 |
| FJ882099 | MSV-A [MZ_Pem5_Moz41_2007] | 2007 |
| FJ882101 | MSV-A [MZ_Xai1_xaimoz_2007] | 2007 |
| FJ882128 | MSV-A [ZA_Pot1_Riz48_2007] | 2007 |
| EU628573 | MSV-A [ZA_Omr_g221_2007] | 2007 |
