# Supplementary material for: Symptom evolution following the emergence of maize streak virus
Source: eLife. 2020 Jan 15;9:e51984. doi: 10.7554/eLife.51984 (PMC7034976; doi:10.7554/eLife.51984)
Supplement: Supplementary file 4. [file elife-51984-supp4.docx]

**Supplementary File 4**. Table: Dataset properties, numbers of generations used and standard deviations (SD) of split frequencies achieved during Bayesian inference of ancestral sequences.

| **Ancestor** | **Number of sequences in alignment** | **Alignment File** | **Independent run** | **Number of generations** | **SD of split frequencies** |
| --- | --- | --- | --- | --- | --- |
| A0 | 252 | SI File S1 | 1  2 | 20.1M  42.4M | 0.037  0.023 |
| A1 | 252 | SI File S1 | 1  2 | 19.7M  27.4M | 0.009  0.007 |
| A2 | 346 | SI File S2 | 1  2  3 | 31.6M  33.8M  41.3M | 0.024  0.020  0.018 |
| A3 | 346 | SI File S2 | 1  2 | 29.7M  37.4M | 0.020  0.015 |
| A4 | 321 | SI File S3 | 1  2 | 44.6M  30.6M | 0.038  0.064 |
| A5 | 346 | SI File S2 | 1  2  3 | 19.3M  28.5M  35.7M | 0.028  0.017  0.014 |
| A6 | 346 | SI File S2 | 1  2 | 14.2M  32.1M | 0.047  0.019 |
